# Comparison of Duration of Response vs Conventional Response Rates and Progression-Free Survival as Efficacy End Points in Simulated Immuno-oncology Clinical Trials

**DOI:** 10.1001/jamanetworkopen.2021.8175

**Published:** 2021-05-28

**Authors:** Chen Hu, Meihua Wang, Cai Wu, Heng Zhou, Cong Chen, Scott Diede

**Affiliations:** 1 Sidney Kimmel Comprehensive Cancer Center, Johns Hopkins University School of Medicine, Baltimore, Maryland; 2 Merck & Co Inc, Kenilworth, New Jersey

## Abstract

**Importance:**

Phase 2 trials and early efficacy end points play a crucial role in informing decisions about whether to continue to phase 3 trials. Conventional end points, such as objective response rate (ORR) and progression-free survival (PFS), have demonstrated inconsistent associations with overall survival (OS) benefits in immune checkpoint inhibitor (ICI) trials. Restricted mean duration of response (DOR) is a rigorous metric that combines both response status and duration information. However, its utility in clinical development has not been comprehensively explored.

**Objective:**

To determine whether using restricted mean DOR in phase 2 trials can advance promising regimens to phase 3 trials sooner and eliminate unfavorable regimens earlier and with a higher degree of confidence compared with PFS and ORR.

**Design, Setting, and Participants:**

This simulated modeling study randomized phase 2 screening trials by resampling 1376 patients from 2 completed randomized phase 3 trials of ICIs. Data were analyzed from August 2019 to July 2020.

**Exposures:**

Use of ICIs.

**Main Outcomes and Measures:**

Restricted mean DOR, PFS, ORR, and OS were estimated and compared between groups. Three scenarios were considered: (1) significant differences in OS, PFS, and ORR; (2) significant differences in OS and noticeable differences in ORR but not PFS; and (3) no differences in OS, PFS, or ORR. For each setting, 5000 randomized phase 2 trials with different sample sizes were simulated, with additional censoring applied to mimic staggered accruals and ensure fair comparisons between different analysis methods. Probabilities of concluding positive phase 2 trials using PFS, ORR, and DOR were summarized and compared.

**Results:**

The restricted mean DOR difference correctly estimated a positive OS benefit more frequently than did the ORR or PFS tests, across different sample sizes, significance levels, and censoring levels evaluated. When both OS and PFS differed, the ranges of true-positive or power rates were 79.2% to 98.7% for DOR, 56.3% to 93.2% for PFS, and 67.0% to 96.0% for ORR. When OS differed but PFS did not, the ranges of power rates were 24.0% to 76.0% for DOR, 3.0% to 19.0% for PFS, and 10.5% to 38.0% for ORR. When OS was similar, the false-positive rate of restricted mean DOR test was close to the chosen significance level.

**Conclusions and Relevance:**

These findings suggest that restricted mean DOR in randomized phase 2 trials is potentially more sensitive and useful than PFS and ORR in estimating the subsequent phase 3 conclusions and, thus, may be considered to complementarily facilitate decision-making in future clinical development.

## Key Points

**Question:**

Does duration of response (DOR), a rigorous metric combining both tumor responses and duration, better inform decisions about continuing or discontinuing before starting phase 3 immuno-oncology clinical trials?

**Findings:**

This simulated modeling study, which was based on simulated randomized phase 2 trials that resampled patients from completed randomized phase 3 trials of immune checkpoint inhibitors, found that restricted mean DOR consistently outperformed progression-free survival and objective response rate in correctly estimating positive overall survival benefits without inflating type I errors.

**Meaning:**

These findings suggest that restricted mean DOR may be a sensitive and informative early efficacy end point in randomized phase 2 trials in immuno-oncology.

## Teaser

This simulated modeling study examines whether using restricted mean duration of response in phase 2 trials can better inform decisions about continuing or discontinuing before starting phase 3 immuno-oncology clinical trials compared with progression-free survival and objective response rate.

## Introduction

Response Evaluation Criteria In Solid Tumors,^1^ a set of qualitative evaluation criteria based on radiographic changes in tumor lesions, has been used widely to inform physicians whether tumors have complete response (CR), partial response (PR), stable disease, or progressive disease. Under the assumption that meaningful changes in tumor response may inform disease prognosis and patient survival, objective response rate (ORR) and progression-free survival (PFS) are used widely in phase 2 oncology trials to inform whether a subsequent phase 3 trial should follow.

Compared with cytotoxic or cytostatic agents, immune checkpoint inhibitors (ICIs) feature unique patterns of tumor response, such as delayed response, durable response, pseudo progression, and hyperprogression.^2^ Moreover, overall survival (OS) benefits have been observed in both the presence and absence of PFS or ORR benefits in ICI trials.^3,4,5^ Collectively, ORR or PFS could be inadequate to capture the tumor response complexity sufficiently and, thus, suboptimal to efficiently inform immuno-oncology clinical development.

Recognizing these shortcomings, clinical trialists increasingly include duration of response (DOR) as a secondary or exploratory end point in ICI trials. DOR is defined as the interval from response initiation (when either CR or PR is first determined) to progression or death, whichever occurs first. Because only a fraction of patients respond to active treatments, analysis of DOR has been limited to descriptive analyses of responders only (eg, Kaplan-Meier [KM] curves of responders). Recently, using well-established statistical methods related to restricted mean survival time (RMST),^6^ Huang et al^7,8^ publicized a conceptually simple approach, restricted mean DOR, to analyze DOR regardless of response status. To the best of our knowledge, restricted mean DOR has been used only in limited clinical trials,^9^ and its utility has not been explored comprehensively.

Phase 2 oncology trials are no longer dominated by single-group designs, largely because of heighted concerns about how reliable they are to screen truly efficacious regimens when historical controls are unreliable or even unavailable. Randomized phase 2 screening design^10^ has been increasingly used and is particularly favored when historical control data are highly uncertain.^11^ This design features a concurrent control group, the use of tumor response–related end points, a large targeted effect size (eg, an extremely strong signal), and a more relaxed type I error control (eg, α = .10).

Using similar analytical tools as Huang et al^8^ and the quality-adjusted time without toxicity and symptoms (Q-TWiST) method,^12^ our overarching goal was to determine whether restricted mean DOR, as well as restricted mean duration of CR (DOCR) or duration of PR (DOPR), could be used as valuable efficacy metrics in early-phase clinical development to better inform decisions about whether to continue to a phase 3 trial. Ideally, a good early efficacy metric should (1) have a high (eg, >80% or 90%) probability of being positive in properly sized randomized phase 2 trials when the OS is indeed positive and (2) have a low probability (eg, purely due to chance) of being positive in phase 2 trials when OS is negative. Accordingly, we simulated randomized phase 2 screening trials by resampling completed randomized phase 3 trials of ICIs and compared the phase 2 findings based on restricted mean DOR with ORR and PFS.

## Methods

This study was not submitted to an institutional review board for approval. Informed consent was not sought because it used deidentified data, in accordance with 45 CFR §46.

### Restricted Mean DOR, DOCR, and DOPR

We first review RMST because it is highly relevant to restricted mean DOR. RMST is a model-free metric summarizing a failure time, such as OS and PFS, and can be estimated by the area under its KM curve.^6^ The use of a restricted mean instead of a straightforward mean is for mathematical reasons when censoring is present. In practice, RMST and related restricted mean times are calculated over a prespecified and clinically meaningful duration (τ), such as 5 years for OS or 12 months for PFS, and can be interpreted as the mean OS time or PFS time up to τ.

**Figure 1.**
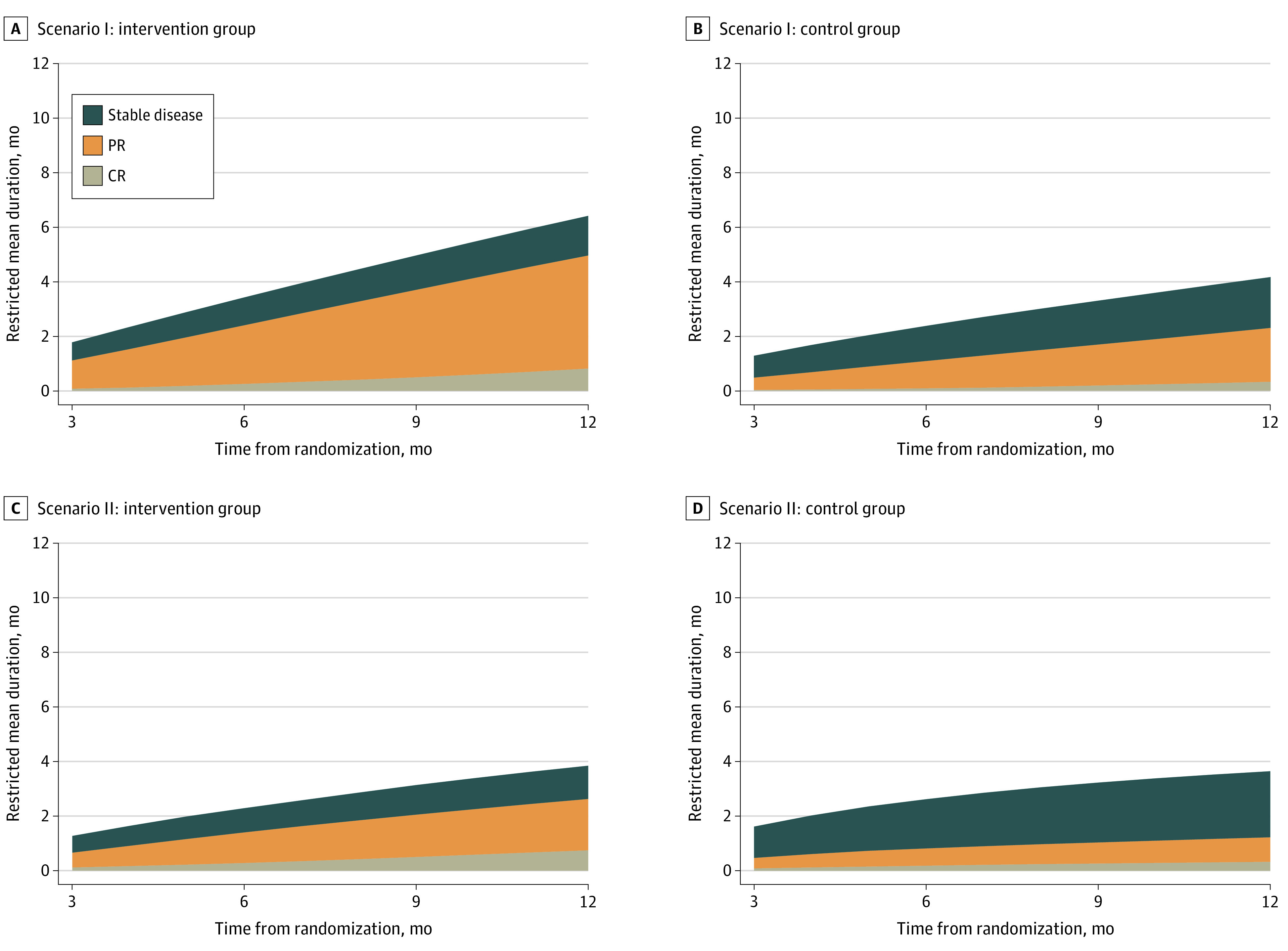
Restricted Mean Time of Progression-Free Survival Partitioned Into Restricted Mean Durations of Complete Response (CR), Partial Response (PR), and Stable Disease Evaluated Up to Different Follow-up Times (3 to 12 Months) The sum of restricted mean durations of CR and PR is restricted mean duration of response. Panel A shows restricted mean durations of CR, PR, and stable disease in immune checkpoint inhibitor and placebo groups of scenario I. Panel B shows restricted mean durations of CR, PR, and stable disease in immune checkpoint inhibitor and placebo groups of scenario II.

Recently, Huang et al^7,8^ publicized the use of restricted mean DOR, which can be calculated by the difference between the KM curve of PFS and KM curve of progression, death, or response event–free time, and is implemented in an R package PBIR^13^ for estimation and statistical inference. Restricted mean DOR is also closely associated with the Q-TWiST method,^14,15^ which partitions RMST of overall survival into 3 distinct states: restricted mean time with toxicity or symptoms, restricted mean TWiST, and restricted mean time relapsed. When patients remain progression free, their response statuses over time are CR, PR, or stable disease, and one can similarly partition restricted mean PFS into states of durations of CR, PR, and stable disease using the Q-TWiST method.^12^ Such a Q-TWiST partition allows one to obtain restricted mean DOCR, DOPR, and duration of stable disease and to alternatively calculate restricted DOR by summing restricted the mean DOCR and DOPR (Figure 1) and is used in this article.

In addition to their intuitive and clinically meaningful interpretation, restricted mean DOR, restricted mean DOCR, and restricted mean DOPR are means of all patients instead of the responders only. This feature allows us to obtain the treatment effect from randomized clinical trials using the difference or ratio between 2 groups. The choice of whether to use the difference or the ratio of DOR to best describe any putative treatment effect is dependent on the choice of τ and the need for interpretation, both of which are beyond the scope of this article. Our main focus here is to determine whether restricted mean DOR, DOCR, or DOPR is useful to improve the decision-making process based on randomized phase 2 trials. In this setting, our understanding of tumor response dynamics could remain limited, and it could be challenging to prospectively determine τ at the design and even analysis stage of randomized phase 2 trials. Therefore, we used the ratios of restricted mean DOR, DOCR, and DOPR to allow a more standardized comparisons (vs their differences) across different choices of τ. We note that the hypothesis-testing results based on difference and ratio of restricted mean DOR are same because both tests can be equivalently converted from the asymptotic distribution of restricted mean DOR.

### Phase 3 Trial Data

To comprehensively evaluate the utility of restricted mean DOR, DOCR, and DOPR, we identified 2 completed, multinational, open-label, active-controlled randomized phase 3 clinical trials of ICI in metastatic solid tumors. Study 1 (ClinicalTrials.gov identifier NCT01866319)^16^ randomized 834 patients from 2013 to 2014 in a 1:1:1 ratio to receive 1 of 2 dose schedules of ICI (doses 1 and 2) or active control as first-line treatment. Study 2 (ClinicalTrials.gov identifier NCT02256436)^17^ randomized 542 patients from 2014 and 2015 equally to receive either ICI or chemotherapy control as second-line treatment; thus, a total of 1376 patients were randomized. They represent 3 scenarios of interest, as summarized in the Table: (1) significant differences in OS, PFS, and ORR, where the proportional hazard assumption is roughly met for PFS; (2) significant differences in OS and noticeable differences in ORR but no significant differences in PFS, where the proportional hazard assumption is violated for PFS and KM curves cross at approximately 6 months; and (3) no clinically meaningful difference in OS, PFS, or ORR.

**Table.** Result Summary of Completed ICI Phase 3 Trials

Outcome	Scenario I, study 1		Scenario II, study 2		Scenario III, study 1	
	ICI (dose 1 + 2)	Control	ICI	Control	ICI dose 1	ICI dose 2
Overall survival						
Median (95% CI)	32.7 (24.5 to 41.6)	15.9 (13.3 to 22.0)	10.3 (8.0 to 11.8)	7.4 (6.1 to 8.3)	31.1 (22.1 to 45.9)	34.2 (23.5 to 42.7)
HR (95% CI)	0.73 (0.61 to 0.88)		0.73 (0.59 to 0.91)		1.00 (0.80 to 1.25)	
Progression-free survival						
Median (95% CI)	8.4 (6.6 to 11.3)	3.4 (2.9 to 4.2)	2.1 (2.0 to 2.2)	3.3 (2.3 to 3.5)	8.4 (5.6 to 13.7)	9.7 (5.8 to 12.0)
HR (95% CI)	0.57 (0.48 to 0.67)		0.98 (0.81 to 1.19)		0.99 (0.81 to 1.20)	
Objective rate response						
Rates, %	33.3 (29.4 to 37.3)	11.9 (8.3 to 16.3)	21.1 (16.4 to 26.5)	11.4 (7.9 to 15.8)	33.7 (28.2 to 39.6)	32.9 (27.4 to 38.7)
Difference, % (95% CI)	21.4 (15.7 to 26.6)		9.6 (3.5 to 15.9)		0.8 (−7.0 to 8.6)	

Abbreviations: HR, hazard ratio; ICI, immune checkpoint inhibitor.

### Resampling Simulations

To simulate a randomized phase 2 screening trial, we randomly sampled individual patients with replacement from a completed phase 3 trial. Randomized, 2-group, phase 2 trials were simulated with 50 or 100 patients in either experimental ICI group or control group (eg, equal randomization). In the absence of specific study hypothesis and settings, the sample sizes of randomized phase 2 trials we used here (eg, 100 or 200 participants) were not chosen on the basis of rigorously sample size justifications. Instead, we chose them to represent the range of sample sizes that a typical randomized phase 2 trial would use. The sample size of 100 participants represents the situation where an initial efficacy evaluation can be sought on the basis of limited resources or rapid readout, and commitments of subsequent phase 3 trials are contingent on observing a significant treatment effect of ORR, PFS, or restricted mean DOR from such a small randomized phase 2 trial. A sample size of 200 covers the situations where a decision of whether to continue or discontinue based on ORR, PFS, or restricted mean DOR can be comfortably drawn with moderate resource commitment (eg, for locally advanced diseases or integrated phase 2 or 3 design).^18^

For each setting, 5000 replicates of randomized phase 2 trials were performed. In each replicate of a simulated trial, we compared 2 groups with (1) log-rank test for PFS; (2) χ^2^ test for ORR; (3) RMST ratio test of PFS at 6, 9, and 12 months; and (4) ratio tests of DOR, DOCR, and DOPR. The first 2 tests were used because of their popularity in practice, and RMST test of PFS was used because of its increasing use in immuno-oncology, especially when nonproportional hazard issues arise.

### Statistical Analysis

A 2-sided α of .05 and .10 was used to claim a positive outcome in the resampled phase 2 trials. Because the completed phase 3 trial had mature follow-up, to appropriately assess the impacts of limited follow-ups and staggered accrual that arise in phase 2 settings, additional censoring following a uniform distribution up to τ was used to simulate phase 2 trials, such that we were able to have fair comparisons between methods based on the number of events (eg, log-rank test of PFS and ORR difference) and follow-up durations (eg, RMST of PFS and restricted mean DOR) in real applications.

Regarding the operating characteristics in phase 2 to phase 3 decision-making, we focused on the proportions of positive phase 2 trials (under 2-sided significance level of .05 or .10). Under scenarios 1 and 2 where the corresponding phase 3 trial was positive according to OS, this proportion may be viewed as a true-positive rate or power, where a higher proportion (eg, >80% or 90%) suggests that the corresponding early efficacy metric is more sensitive to estimate meaningful OS differences. Under scenario 3, where there is a lack of difference in OS between 2 ICI doses, this proportion may be viewed as a false-positive rate or type I error; when it is close to the α level used (eg, 5% or 10% as chosen), it suggests that the corresponding early efficacy metric is uninformative and any false-positive finding is purely due to chance. An ideal early efficacy metric would have a high chance to identify positive signals of OS when there it truly exists, but not overly report the false positives.

Because of the relatively small sample sizes used in resampled phase 2 trials, especially when there were 50 participants per group, we used bootstrap resampling and permutation tests to obtain the associated 95% CI and *P* values. Statistical analysis was performed using R statistical software version 3.5.1 (R Project for Statistical Computing). Data were analyzed from August 2019 to July 2020.

## Results

### Partition Restricted Mean PFS and DOR

We first illustrate how partitioning restricted mean PFS into restricted mean DOCR, DOPR, and duration of stable disease may reveal additional insights of tumor responses, as shown in Figure 1. For example, in scenario I (Figure 1A), the restricted mean PFS for ICI group, up to 12 months, is 6.4 months. Of the 6.4-month restricted mean PFS, the restricted mean DOR is 4.9 months, which includes a restricted mean DOCR of 0.8 month and restricted mean DOPR of 4.1 months. In contrast, for the control group, the restricted mean PFS up to 12 months is 4.2 months, which includes a restricted mean DOR of 2.3 months, restricted mean DOCR of 0.3 month, and restricted mean DOPR of 2.0 months. By contrasting these partitions over a range of τ (eg, 3-12 months), it is apparent that the prolonged PFS in the ICI group was largely associated with a longer restricted mean DOR (longer restricted DOCR and DOPR). Moreover, in scenario II (Figure 1B), where the distribution and restricted mean of PFS were not meaningfully different between groups, restricted mean DOR (both DOCR and DOPR) in ICI group was still meaningfully longer than control group.

### Resampling Simulations

**Figure 2.**
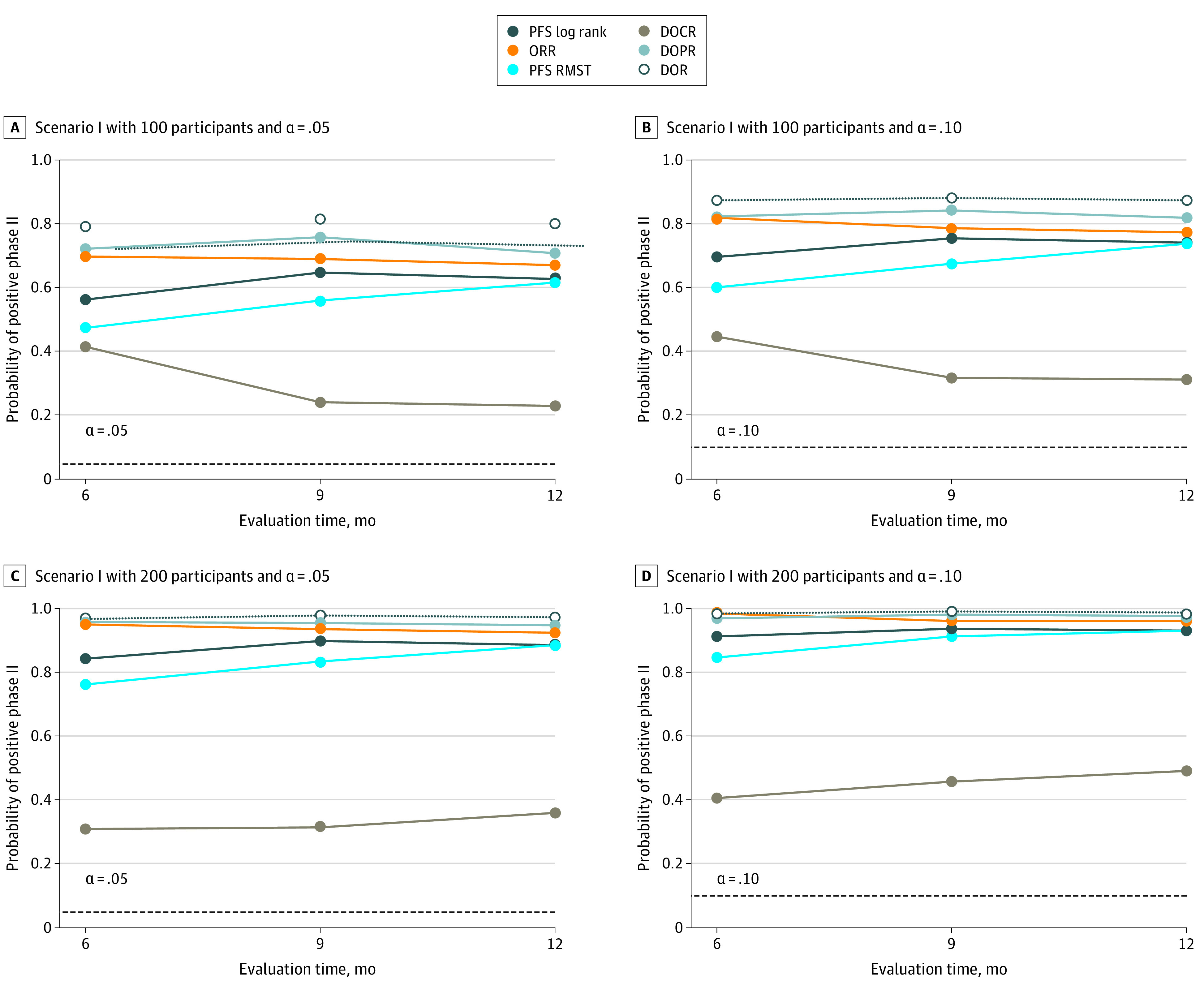
Results Resampling Simulations for Scenario I: Proportion of Claiming Positive (Rejecting Null Hypothesis) Using Respective Tests and Evaluation Time The proportions of censoring were 42%, 39%, and 34% when the evaluation time was 6, 9, and 12 months, respectively. DOCR indicates duration of complete response; DOPR, duration of partial response; DOR, duration of response; ORR, objective response rate; PFS, progression-free survival; RMST, restricted mean survival time.

**Figure 3.**
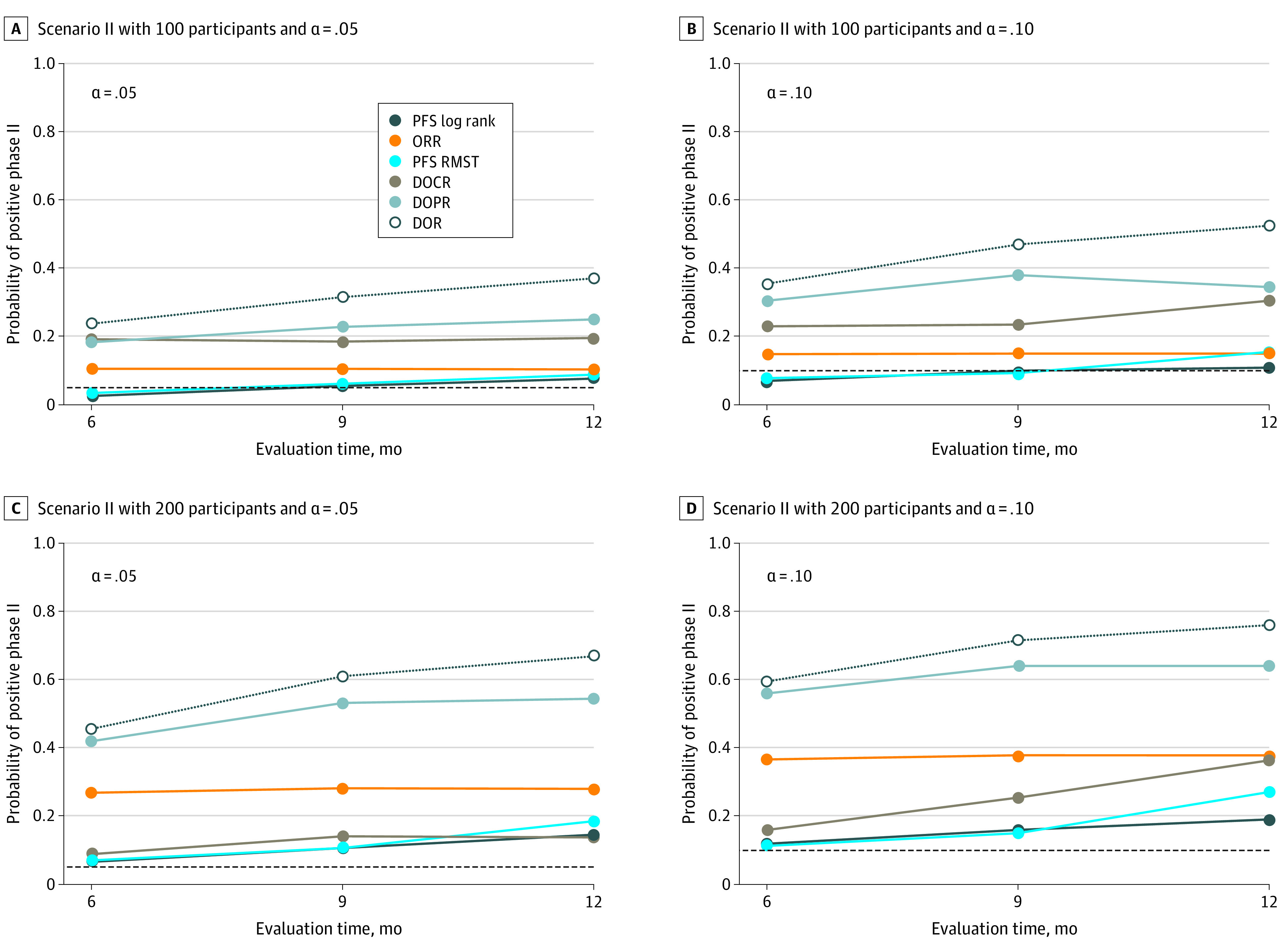
Results of Resampling Simulations for Scenario II: Proportion of Claiming Positive (Rejecting Null Hypothesis) Using Respective Tests and Evaluation Time The proportions of censoring were 26%, 20%, and 16% when evaluation time was 6, 9, and 12 months, respectively. DOCR indicates duration of complete response; DOPR, duration of partial response; DOR, duration of response; ORR, objective response rate; PFS, progression-free survival; RMST, restricted mean survival time.

On the basis of 5000 simulated randomized phase 2 trials with 100 or 200 participants, Figure 2 and Figure 3 summarize the probabilities of being positive at a 2-sided significance level of .05 or .10. For example, for 100 participants with 2-sided α = .05, under scenario I (Figure 2 and eTable 1 in the Supplement), the range of true-positive rates or powers of restricted mean DOR test at τ of 6 to 12 months is 79.2% to 98.7%, which is higher than that of PFS log-rank test (56.3%-93.2%), restricted mean PFS test (47.5%-93.3%), and χ^2^ test of ORR (67.0%-96.0%). Such findings held across different sample sizes, follow-up times (τ), and type I errors. More remarkable findings were observed under scenario II (Figure 3 and eTable 2 in the Supplement), when OS differed but the KM curves of PFS crossed at approximately 6 months. The log-rank test failed to detect any difference because of the nonproportional hazard issue, and so did the RMST test of PFS (3.0%-19.0%). The power to detect ORR differences was also too low to be practically usable even with 200 participants (10.5%-38.0%). Meanwhile, the ranges of power rates were 24.0% to 76.0% for DOR.

**Figure 4.**
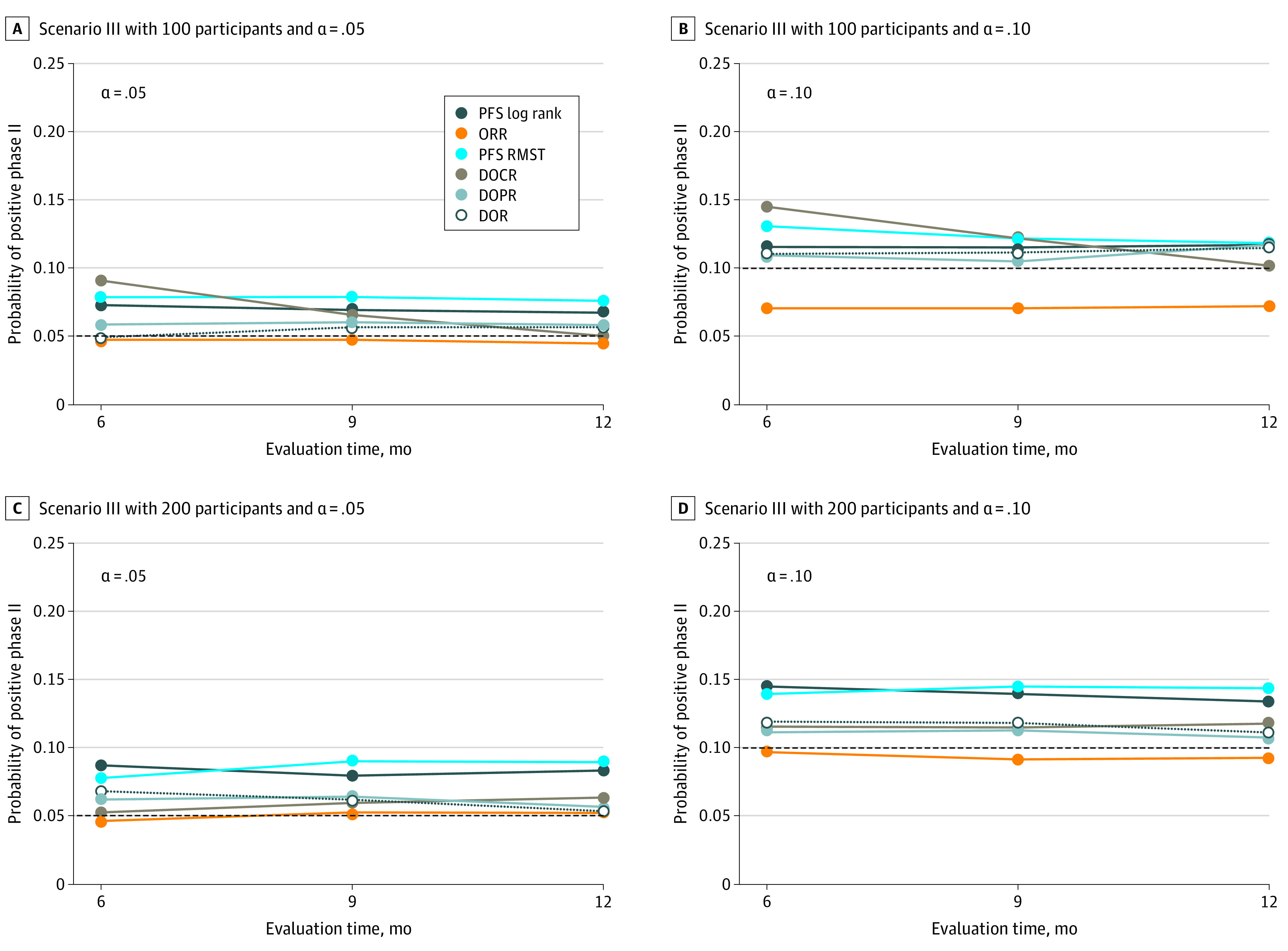
Results of Resampling Simulations for Scenario III: Proportion of Claiming Positive (Rejecting Null Hypothesis) Using Respective Tests and Evaluation Time The proportions of censoring were 52%, 49%, and 45% when the evaluation time was 6, 9, and 12 months, respectively. DOCR indicates duration of complete response; DOPR, duration of partial response; DOR, duration of response; ORR, objective response rate; PFS, progression-free survival; RMST, restricted mean survival time.

When there is lack of OS differences (scenario III in Figure 4 and eTable 3 in the Supplement), across all sample sizes and significance levels, the probabilities of claiming positive findings on the basis of restricted mean DOR were reasonably close to the α level used. The false-positive rates of the ORR test were noticeably lower than the α level when the sample size was 100 and α = .05, whereas that of PFS log-rank test or RMST tended to be slightly larger than the α level used. Practically speaking, however, none of the early efficacy metrics would likely proceed into phase 3 stages noticeably more frequently than by chance.

When τ increased and censoring proportion decreased, we found that the powers of PFS log-rank test and RMST test both increased as more PFS events accumulated. The powers of restricted mean DOR test and ORR difference were not sensitive to the choice of τ and censoring proportions, which may be useful if τ is difficult to choose.

Of note, the performance of restricted mean DOR always outperformed that of DOCR or DOPR, possibly because of the minimal contribution of restricted mean DOCR in the setting we investigated. In light of the satisfactory performance of restricted mean DOR, the utility of restricted mean DOCR and DOPR in informing decision-making becomes limited. Nonetheless, the analytical tools of partitioning may be useful at least in descriptive analyses and may be useful in settings where nontrivial proportion of CR is anticipated.

## Discussion

The purpose of this work is to use duration of tumor response to better inform the decision-making in clinical development in the era of immuno-oncology. According to our simulations, restricted mean DOR has a higher power than ORR and PFS (log-rank or RMST test) in phase 2 trials, when OS is indeed different, and makes false-positive claims similar to the type I error used. To the best of our knowledge, these resampling simulations provide the first evidence suggesting that restricted mean DOR in randomized phase 2 trials has the potential to improve high-stake decision-making about whether to continue or discontinue trials in clinical development.

Importantly, the use of restricted mean DOR overcomes a long-standing challenge—that is, DOR is only analyzed among responders. In contrast, restricted mean DOR is based on both responders and nonresponders, such that we can make valid conclusions based on all intent-to-treat patients. It naturally synthesizes the potential benefit in ORR, PFS, or time to response, even when ORR and PFS are similar but time to response differs between groups. Therefore, it is particularly suitable to simplify decision-making and bypass potential challenges due to multiple comparisons. Although our focus in this article centers on decision-making using restricted mean DOR, we note that its interpretation should make itself a routine secondary end point in oncology clinical trials. Exploiting the association between restricted mean DOR and DOCR and DOPR may be of interest when a deeper exploration of tumor response dynamics is desired.

Like RMST methods, the choice of τ is an important aspect when using restricted mean DOR in practice but is beyond the scope of this article. Generally speaking, τ should be chosen according to clinical interpretation and knowledge and should also be context specific. It should be long enough to allow meaningful differences in both the depth and duration of responses, but also short enough to make rapid readout feasible. Interested readers may refer to Tian et al^19^ and the appendix in the article by Huang et al,^8^ which provide useful recommendations on selecting τ to mimic the data-driven window for log-rank test and hazard ratio.

### Limitations

This study has several limitations. As the first step to evaluate how useful restricted mean DOR is in decision-making, we focused solely on how well restricted mean DOR difference estimated the OS benefit. In practice, however, the decision-making is far more complex and comprehensive. The overall benefit-risk profile is far more important than prolonging OS only; restricted mean DOR itself is a meaningful end point, making it more useful than merely a surrogate end point.

In addition, data from only 2 real trials were used. However, these trials are representative ICI trials, and we believe the use of restricted mean DOR, including but not limited to our results, are not affected by the trial-specific information. In fact, as the clinical development of ICI expands to ICI combination and ICI monotherapy becomes the standard of care, more research should be performed to evaluate whether our findings hold in more diseases and in more contemporary settings.

Another limitation is that the study focused on the use of restricted mean DOR in randomized phase 2 screening design. Other phase 2 designs are of use and interest in practice, such as single-group expansion cohorts^20^ and randomized pick-the-winner selection designs.^21^ When restricted mean PFS and DOR can be reliably summarized using historical controls, additional investigations in these phase 2 designs are certainly needed.

## Conclusions

On the basis of resampled phase 3 trials, this simulated modeling study demonstrated that restricted mean DOR in randomized phase 2 trials is potentially more sensitive and useful than PFS and ORR to estimate the subsequent phase 3 conclusions. Therefore, we recommend that clinical trialists and investigators routinely report restricted mean DOR and use it to better inform high-stake clinical development decision-making.

## Notes

Supplement. **eTable 1.** Numeric Results of Resampling Simulations for Scenario I **eTable 2.** Numeric Results of Resampling Simulations for Scenario II **eTable 3.** Numeric Results of Resampling Simulations for Scenario III

## References

[1] Eisenhauer EA, Therasse P, Bogaerts J, . New response evaluation criteria in solid tumours: revised RECIST guideline (version 1.1). Eur J Cancer. 2009;45(2):228-247. doi:10.1016/j.ejca.2008.10.026 19097774

[2] Wang Q, Gao J, Wu X. Pseudoprogression and hyperprogression after checkpoint blockade. Int Immunopharmacol. 2018;58:125-135. doi:10.1016/j.intimp.2018.03.018 29579717

[3] Huang B. Some statistical considerations in the clinical development of cancer immunotherapies. Pharm Stat. 2018;17(1):49-60. doi:10.1002/pst.1835 29098766

[4] Robert C, Schachter J, Long GV, ; KEYNOTE-006 Investigators. Pembrolizumab versus ipilimumab in advanced melanoma. N Engl J Med. 2015;372(26):2521-2532. doi:10.1056/NEJMoa1503093 25891173

[5] Bellmunt J, de Wit R, Vaughn DJ, ; KEYNOTE-045 Investigators. Pembrolizumab as second-line therapy for advanced urothelial carcinoma. N Engl J Med. 2017;376(11):1015-1026. doi:10.1056/NEJMoa1613683 28212060 PMC5635424

[6] Uno H, Claggett B, Tian L, . Moving beyond the hazard ratio in quantifying the between-group difference in survival analysis. J Clin Oncol. 2014;32(22):2380-2385. doi:10.1200/JCO.2014.55.2208 24982461 PMC4105489

[7] Huang B, Tian L, Talukder E, Rothenberg M, Kim DH, Wei LJ. Evaluating treatment effect based on duration of response for a comparative oncology study. JAMA Oncol. 2018;4(6):874-876. doi:10.1001/jamaoncol.2018.0275 29710201 PMC6584319

[8] Huang B, Tian L, McCaw ZR, . Analysis of response data for assessing treatment effects in comparative clinical studies. Ann Intern Med. 2020;173(5):368-374. doi:10.7326/M20-0104 32628533 PMC7773521

[9] Choueiri TK, Motzer RJ, Rini BI, . Updated efficacy results from the JAVELIN Renal 101 trial: first-line avelumab plus axitinib versus sunitinib in patients with advanced renal cell carcinoma. Ann Oncol. 2020;31(8):1030-1039. doi:10.1016/j.annonc.2020.04.010 32339648 PMC8436592

[10] Rubinstein LV, Korn EL, Freidlin B, Hunsberger S, Ivy SP, Smith MA. Design issues of randomized phase II trials and a proposal for phase II screening trials. J Clin Oncol. 2005;23(28):7199-7206. doi:10.1200/JCO.2005.01.149 16192604

[11] Jemielita T, Tse A, Chen C. Oncology phase II proof-of-concept studies with multiple targets: randomized controlled trial or single arm? Pharm Stat. 2020;19(2):117-125. doi:10.1002/pst.1972 31424631

[12] Glasziou PP, Simes RJ, Gelber RD. Quality adjusted survival analysis. Stat Med. 1990;9(11):1259-1276. doi:10.1002/sim.4780091106 2277877

[13] Luo X, Huang B, Tian L. PBIR: estimating the probability of being in response and related outcomes. Published September 17, 2020. Accessed April 2, 2021. https://cran.r-project.org/web/packages/PBIR/PBIR.pdf

[14] Goldhirsch A, Gelber RD, Simes RJ, Glasziou P, Coates AS. Costs and benefits of adjuvant therapy in breast cancer: a quality-adjusted survival analysis. J Clin Oncol. 1989;7(1):36-44. doi:10.1200/JCO.1989.7.1.36 2642538

[15] Gelber RD, Cole BF, Gelber S, Goldhirsch A. Comparing treatments using quality-adjusted survival: the Q-TWiST method. Am Stat. 1995;49(2):161-169. doi:10.2307/2684631

[16] Schachter J, Ribas A, Long GV, . Pembrolizumab versus ipilimumab for advanced melanoma: final overall survival results of a multicentre, randomised, open-label phase 3 study (KEYNOTE-006). Lancet. 2017;390(10105):1853-1862. doi:10.1016/S0140-6736(17)31601-X 28822576

[17] Bellmunt J, de Wit R, Vaughn DJ, ; KEYNOTE-045 Investigators. Pembrolizumab as second-line therapy for advanced urothelial carcinoma. N Engl J Med. 2017;376(11):1015-1026. doi:10.1056/NEJMoa1613683 28212060 PMC5635424

[18] Korn EL, Freidlin B, Abrams JS, Halabi S. Design issues in randomized phase II/III trials. J Clin Oncol. 2012;30(6):667-671. doi:10.1200/JCO.2011.38.5732 22271475 PMC3295562

[19] Tian L, Jin H, Uno H, . On the empirical choice of the time window for restricted mean survival time. Biometrics. 2020;76(4):1157-1166. doi:10.1111/biom.13237 32061098 PMC8687138

[20] Manji A, Brana I, Amir E, . Evolution of clinical trial design in early drug development: systematic review of expansion cohort use in single-agent phase I cancer trials. J Clin Oncol. 2013;31(33):4260-4267. doi:10.1200/JCO.2012.47.4957 24127441

[21] Rubinstein L, Crowley J, Ivy P, Leblanc M, Sargent D. Randomized phase II designs. Clin Cancer Res. 2009;15(6):1883-1890. doi:10.1158/1078-0432.CCR-08-2031 19276275 PMC3774021

