# Supplementary material for: Comparison of Duration of Response vs Conventional Response Rates and Progression-Free Survival as Efficacy End Points in Simulated Immuno-oncology Clinical Trials
Source: JAMA Netw Open. 2021 May 28;4(5):e218175. doi: 10.1001/jamanetworkopen.2021.8175 (PMC8164100; doi:10.1001/jamanetworkopen.2021.8175)
Supplement: Supplement. — eTable 1. Numeric Results of Resampling Simulations for Scenario I eTable 2. Numeric Results of Resampling Simulations for Scenario II eTable 3. Numeric Results of Resampling Simulations for Scenario III [file jamanetwopen-e218175-s001.pdf]

Supplemental Online Content

Hu C, Wang M, Wu C, Zhou H, Chen C, Diede S. Comparison of duration of response vs conventional response rates and progression-free survival as efficacy end points in simulated immuno-oncology clinical trials. *JAMA Netw Open*. 2021;4(5):e218175. doi:10.1001/jamanetworkopen.2021.8175

eTable 1. Numeric Results of Resampling Simulations for Scenario I

eTable 2. Numeric Results of Resampling Simulations for Scenario II

eTable 3. Numeric Results of Resampling Simulations for Scenario III

This supplemental material has been provided by the authors to give readers additional information about their work.

© 2021 Hu C et al. *JAMA Network Open.*

eTable 1. Numeric Results of Resampling Simulations for Scenario I

Proportions of rejecting the null based on respective test and evaluation time τ are reported. The proportions of censoring are 42%, 39% and 34% when τ=6, 9, and 12 months, respectively. See Figure 2 in the text.

		α=0.05			α=0.1		
Sample Size	Test	τ =6m	τ =9m	τ =12m	τ =6m	τ =9m	τ =12m
N=100	PFS log-rank	56.3%	64.7%	62.9%	69.7%	75.6%	74.3%
	PFS RMST	47.5%	55.9%	61.8%	60.5%	67.6%	74.0%
	ORR χ^2 test	69.7%	68.9%	67.0%	81.5%	78.9%	77.4%
	DOR	79.2%	81.4%	79.8%	87.4%	87.8%	87.1%
	DOCR	41.6%	24.1%	23.0%	44.5%	32.0%	31.3%
	DOPR	72.4%	75.8%	70.7%	82.5%	84.2%	82.1%
N=200	PFS log-rank	84.6%	89.7%	88.6%	91.3%	93.8%	93.2%
	PFS RMST	76.2%	83.2%	88.4%	85.0%	91.4%	93.3%
	ORR χ^2 test	95.2%	93.8%	92.6%	97.3%	96.5%	96.0%
	DOR	96.8%	97.8%	97.3%	98.6%	99.0%	98.7%
	DOCR	31.0%	31.7%	36.3%	40.6%	45.7%	49.0%
	DOPR	95.4%	96.0%	94.7%	97.1%	98.0%	97.4%

© 2021 Hu C et al. *JAMA Network Open.*

eTable 2. Numeric Results of Resampling Simulations for Scenario II

Proportions of rejecting the null based on respective test and evaluation time τ are reported. The proportions of censoring are 26%, 20% and 16% when τ =6, 9, and 12 months, respectively. See Figure 3 in the text.

		α=0.05			α=0.1		
Sample Size	Test	τ =6m	τ =9m	τ =12m	τ =6m	τ =9m	τ =12m
N=100	PFS log-rank	3.0%	5.5%	7.5%	7.5%	10.0%	11.0%
	PFS RMST	3.5%	6.0%	9.0%	8.0%	9.5%	15.5%
	ORR χ^2 test	10.5%	10.5%	10.5%	15.0%	15.0%	15.0%
	DOR	24.0%	31.5%	37.0%	35.5%	47.0%	52.5%
	DOCR	19.5%	18.5%	19.5%	23.0%	23.5%	30.5%
	DOPR	18.5%	23.0%	25.0%	30.5%	38.0%	34.5%
N=200	PFS log-rank	6.5%	10.5%	14.5%	12.0%	16.0%	19.0%
	PFS RMST	7.0%	10.5%	18.5%	11.5%	15.0%	27.0%
	ORR χ^2 test	27.0%	28.0%	28.0%	36.5%	38.0%	38.0%
	DOR	45.5%	61.0%	67.0%	59.5%	71.5%	76.0%
	DOCR	8.5%	14.0%	13.5%	16.0%	25.5%	36.5%
	DOPR	42.0%	53.0%	54.5%	56.0%	64.0%	64.0%

© 2021 Hu C et al. *JAMA Network Open.*

eTable 3. Numeric Results of Resampling Simulations for Scenario III

Proportions of rejecting the null based on respective test and evaluation time τ are reported. The proportions of censoring are 52%, 49% and 45% when τ =6, 9, and 12 months, respectively. See Figure 4 in the text.

		α=0.05			α=0.1		
Sample Size	Test	τ =6m	τ =9m	τ =12m	τ =6m	τ =9m	τ =12m
N=100	PFS log-rank	7.3%	7.0%	6.7%	11.6%	11.5%	11.7%
	PFS RMST	7.9%	7.9%	7.6%	13.1%	12.2%	11.8%
	ORR χ^2 test	4.7%	4.8%	4.5%	7.1%	7.1%	7.2%
	DOR	4.9%	5.7%	5.7%	11.1%	11.2%	11.5%
	DOCR	9.1%	6.6%	5.0%	14.5%	12.2%	10.2%
	DOPR	5.9%	6.0%	5.8%	11.0%	10.5%	11.6%
N=200	PFS log-rank	8.7%	8.0%	8.4%	14.5%	14.0%	13.4%
	PFS RMST	7.8%	9.0%	8.9%	14.0%	14.5%	14.4%
	ORR χ^2 test	4.7%	5.3%	5.3%	9.7%	9.2%	9.3%
	DOR	6.8%	6.2%	5.4%	11.9%	11.9%	11.1%
	DOCR	5.3%	6.0%	6.4%	11.6%	11.5%	11.8%
	DOPR	6.2%	6.4%	5.7%	11.2%	11.3%	10.8%

© 2021 Hu C et al. *JAMA Network Open.*
